# Costs of fracture-related infection: the impact on direct hospital costs and healthcare utilisation

**DOI:** 10.1007/s00068-024-02497-9

**Published:** 2024-04-09

**Authors:** S. Haidari, M.A.S. Buijs, J.D.J. Plate, J. J. Zomer, F.F.A. IJpma, F. Hietbrink, G.A.M. Govaert

**Affiliations:** 1 Department of Trauma Surgery, University Medical Centre Utrecht, Utrecht, The Netherlands; 2 Department of Surgery, Radboud University Medical Centre, Nijmegen, Tilburg The Netherlands; 3 Finance Department, University Medical Centre Utrecht, Utrecht, The Netherlands; 4 Finance Department, Wageningen University and Research, Wageningen, The Netherlands; 5 Department of Trauma Surgery, University Medical Centre Groningen, Groningen, The Netherlands

**Keywords:** Fracture-related infection, Hospital costs, Healthcare utilization, Healthcare costs, Osteomyelitis, Trauma surgery

## Abstract

**Purpose:**

Fracture-Related Infection (FRI) is associated with high medical costs and prolonged healthcare utilization. However, limited data is available on the financial impact. The purpose of this study was to investigate the impact of FRI on direct hospital costs and healthcare utilization.

**Methods:**

This was a retrospective cohort study in a level-1 trauma centre in the Netherlands. Patients ≥ 18 years, after open reduction and internal fixation of a long bone fracture between January 1st 2016 and November 1st 2021, were included. Exclusion criteria were Injury Severity Score (ISS) ≥ 16, indefinable data on costs or incomplete follow-up. Hospital costs related to fracture treatment were individually calculated based on procedure codes raised with a fixed percentage of overhead expenses, in line with hospital billing policies.

**Results:**

In total, 246 patients were included with a median follow-up of 1 year (IQR 0.6–1.8). A total of 45 patients developed FRI, of whom 15 patients had an FRI recurrence. Compared to non-FRI patients, median hospital costs from an FRI patient without and with recurrence, were respectively three (3.1) and seven (7.6) times higher. Compared to non-FRI patients, increased costs in patients with FRI or recurrent FRI are due to respectively a fivefold or even tenfold prolonged length-of-stay, two or seven additional infection-related surgeries, and 21 or 55 days of intravenous antibiotic treatment.

**Conclusion:**

Direct healthcare costs of patients with single occurrence of FRI after long bone fracture treatment are three times higher compared to non-FRI patients. In case of FRI-recurrence, the differences in costs might even increase to sevenfold. To put this in perspective, cost of severely injured trauma patients were recently established at approximately 25.000 euros. Compared to non-FRI patients, increased costs in patients with FRI or recurrent FRI are due to respectively a fivefold or even tenfold prolonged length-of-stay, two or seven additional infection-related surgeries and 21 or 55 days of intravenous antibiotic treatment. Not only from patient perspective but also from a financial aspect, it is important to focus on prevention of (recurrent) FRI.

## Introduction

Previous studies showed that surgical site infection substantially increases the financial burden on total healthcare expenditure, mostly driven by the requirement of additional surgeries, extended hospitalisations, re-admissions and extensive postoperative antibiotic treatment regime [1–3]. With healthcare systems under rising economic pressure, there is an increasing interest in measures to reduce costs. However, compared to surgical site infection in general, studies solely focusing on the impact of fracture related infection (FRI) on healthcare utilisation and costs, are limited. The need for studies focusing on costs of complications after fracture surgery was previously recognized [4], emphasizing the fact that especially fracture care is associated with higher infection rates compared to other surgical procedures [1]. Although several studies analysed the socio-economic impact of FRI and stated that direct hospital-related costs are up to eight times higher compared to that of non-infected fractures [5], there is still a lot of unclear information. Most of these studies were heterogeneous, included only small numbers of orthopaedic trauma patients (*N* = 7) [6] or were limited to one fracture type and therefore not generalizable to clinical practice [7, 8]. Due to an aging population, the number of fracture related surgeries is further increasing and thus the prevalence of FRI is expected to rise [9]. In an era with major healthcare budgetary challenges, it is important to gain more insight in the costs associated with FRIs.

The main objective of this study was to investigate the impact of FRI on direct hospital costs and healthcare utilization compared to non-FRI patients.

## Methods

### Study design and setting

This was a cohort study based on retrospectively collected data of patients who underwent surgery for fracture of a long bone (femur, tibia, fibula, humerus, forearm) in a level-1 trauma centre in the Netherlands, The University Medical Centre Utrecht (UMCU). The medical ethical committee of the University Medical Centre Utrecht reviewed the study protocol and granted a waiver (reference METC 20 − 004/C).

### Patient population

All patients, ≥ 18 years old, who underwent open reduction and internal fixation (ORIF) for fracture of a long bone (femur, tibia, fibula, humerus, forearm) from January 1st 2016 to November 1st 2021 were included in this study. Patients were identified by operative procedure codes of ORIF of long bone fractures. Patients were excluded from participation if they had multiple injuries (defined as an Injury Severity Score (ISS) ≥ 16 [10]), periprosthetic or pathologic fractures, if ORIF was combined or related to arthroplasty, if healthcare costs were indefinable or in case of inadequate follow-up. Costs were deemed indefinable if they consisted of inseparable comorbidity-related costs or if costs were incomplete due to recent missing declarations. Follow-up had to be at least until consolidation of the fracture and, in case of FRI, six months after the cessation of both surgical and antibiotic therapy without any signs of recurrence. In case of discharge from further follow-up all patients received strict instructions to return to our hospital if any signs of infection (re-)occurred.

### Variables

FRI was diagnosed according to the confirmative FRI consensus criteria: the presence of at least two phenotypically identical pathogens identified in two deep separate tissue cultures, purulent drainage or the presence of pus and/or the presence of a fistula, a sinus or wound breakdown [11]. Recurrence of FRI was defined as re-appearance of confirmatory criteria after cessation of surgical and antimicrobial treatment of the initial infection. Other collected variables were grouped in patient (gender, age, BMI, smoking status, ASA score, Charlson Comorbidity Index [12]), trauma (mechanism, high-energy, crush, Injury Severity Score [10]), fracture (location, AO/OTA classification [13], soft tissue status) and operation characteristics (type of main implant, direct closure, implant removal). Open fractures were classified by the Gustilo-Anderson Classification [14]. All variables from patients with FRI were retrieved from the UMCU FRI database (compiled in the electronic data capturing program CASTOR) and, if necessary, supplemented with data from the medical files. From patients without FRI, all data was extracted from the medical files.

### Outcomes

The main study outcome was direct fracture related healthcare costs, which was defined as all fracture treatment related hospital costs from first presentation until discharge from follow-up. Costs were based on individually declared fracture treatment related procedure codes and gathered in collaboration with the hospital financial department. The procedure codes were grouped per patient in five hospital related cost categories: hospitalisation, surgery, consults, imaging and other. Hospitalisation costs were calculated based on an average day-based care fee multiplied by the patient’s actual length-of-stay (LOS). Other day-based fees were applicable for intensive care or day-care admissions. Surgical costs consisted of material-related costs (i.e. implants, sutures), the surgeons honoraria and facility related costs (i.e. operation room rental fee, operation assistants fee). Facility related costs were based on a standard fee per operative procedure within a certain timeframe. In case of an extensive procedure, based on operation time, more facility related costs were taken into account. The consults category involved all trauma related medical visits, including visits to the outpatient clinic, emergency room or other involved specialties, such as the rehabilitation physician or physiotherapist. Imaging concerned all injury, fracture and complication related radiological and nuclear imaging examinations from presentation until discharge from follow-up. Other costs were mainly defined by laboratory and microbiology costs and outpatient costs related to wound treatment and (construction or removal of) casts. The sum of five categories was interpreted as the total amount of clinical costs per patient. A fixed hospital-based percentage of 30% was defined by the financial department and added as overhead expenses, this is custom practice for all cost-calculations in the UMCU. Costs could not be presented in euros since exact prices are regarded hospital private information. Therefore, it was necessary to express costs as ratios, indicating the elevated cost relationships between non-FRI, FRI and recurrent FRI patients.

Secondary outcomes were number of additional surgeries, number of additional admissions, duration and costs of antibiotic usage and total LOS. Implant removal was evaluated separately from additional surgeries. Duration and costs of fracture related antimicrobial treatment were calculated separately. Duration of iv and oral antibiotic treatment was retracted from the patient medical files. Calculated costs were based on prices published by Healthcare Institute of the Netherlands (Zorginstituut Nederland, March 2023). Antibiotic treatments of non-fracture related complications were disregarded. Also, costs related to administration of antibiotic treatment were not considered.

### Statistical analyses

Baseline characteristics were analysed descriptively using counts with percentages for categorical variables and mean with standard deviations (SD) in case of a normally distributed continuous variable, otherwise the median with interquartile ranges (IQR) were described. Normality was assessed using histogram and the normal probability plots (Q-Q plots).

Outcomes were compared between patients with FRI and without FRI and analysed descriptively using counts with percentages and medians with IQR.

For statistical evaluation of all data, IBM Statistics for Windows Software (SPSS) was used (version 26.0, Armonk, NY, USA: IBM Corp.).

## Results

### Patient baseline characteristics

**Fig. 1:**
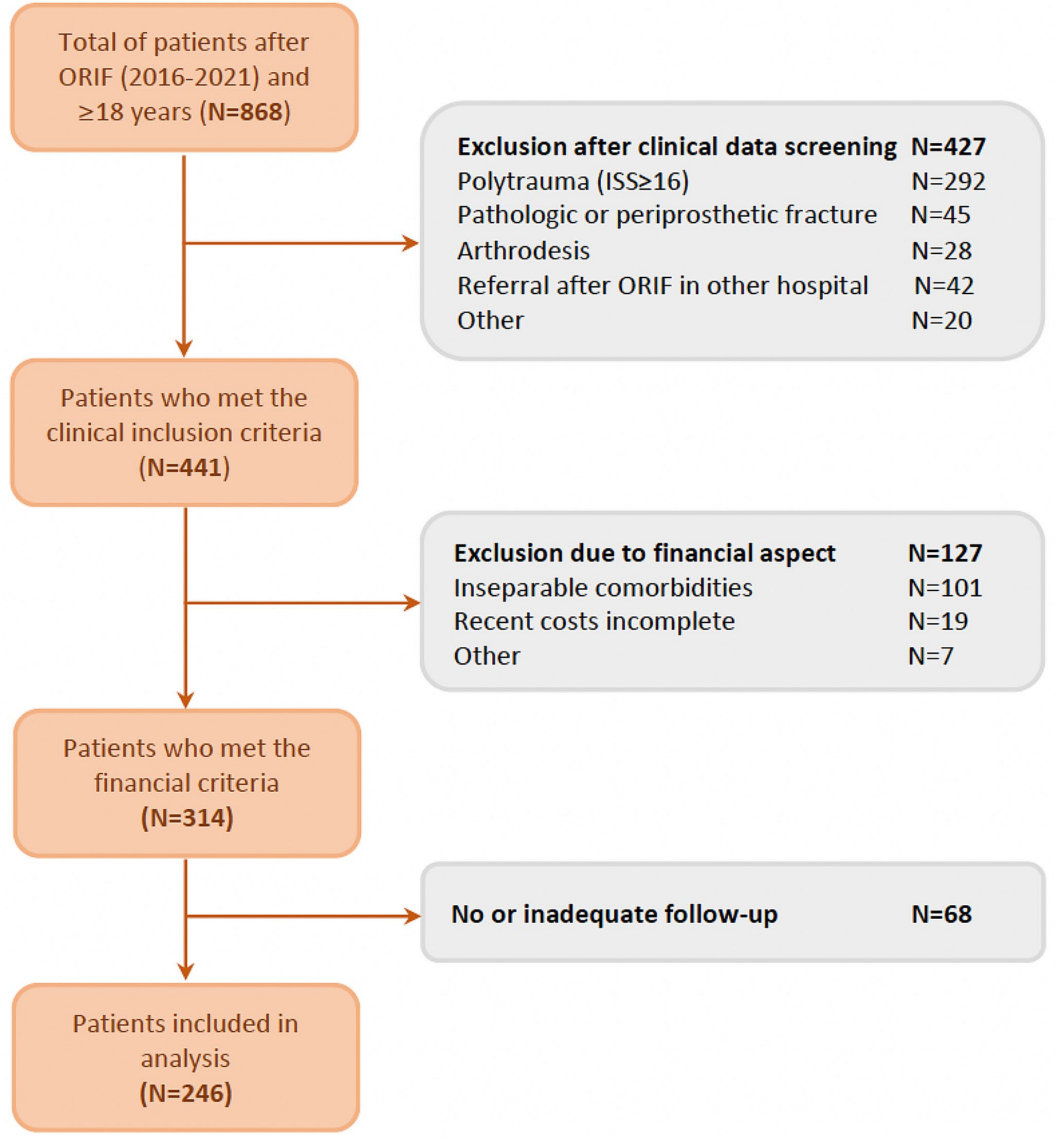
Flowchart patient inclusion

During the study period, 868 patients eligible for inclusion were identified, of which 246 patients met the inclusion criteria and were included in the analysis (Fig. 1). Baseline characteristics of the 246 included patients are presented in Table 1. The majority of patients were male (*N* = 146, 59%), with a median age of 42 years (IQR 27–57) and an ASA score of 1 (*N* = 143, 58%). The median ISS was 9 (IQR 4–9). Most fractures were closed (*N* = 186, 75%) and located at the tibia or fibula (*N* = 115, 46%). Most patients had no additional operations (IQR 0–1) besides implant removal, which was executed in 79 patients (32%). The median total length-of-stay (LOS) was 6 days (IQR 3–14). After ORIF, 45 patients developed an FRI (18%), of whom 15 developed a recurrent FRI (6%). The median follow-up was 1 year (IQR 0.63–1.84).

**Table 1:** Baseline characteristics per group

Criteria	All patients (*N* = 246)	Non-FRI (*N* = 201)	Single FRI (*N* = 30)	FRI recurrence (*N* = 15)
*Patient characteristics*				
Gender (male), N (%)	146 (59)	117 (58)	19 (63)	10 (67)
Age (years), median (IQR)	42 (27–57)	38 (25–55)	57 (42–66)	50 (28–60)
BMI (kg/m^2^), mean (SD) (*N* = 230)	25 (4)	25 (4)	25 (5)	24 (3)
Smoking, N (%) (*N* = 203)	78 (32)	67 (33)	5 (17)	6 (40)
ASA, N (%) *ASA 1* *ASA 2* *ASA 3* *ASA 4*	143 (58) 97 (39) 6 (2) 0	119 (59) 77 (38) 5 (3) 0	16 (53) 13 (43) 1 (3) 0	8 (53) 7 (65) 0 0
CCI (%10-year survival), median (IQR)	98 (96–98)	98 (96–98)	96 (90–98)	96 (90–98)
*Trauma characteristics*				
Trauma mechanism, N (%) *Car* *Motorbike/scooter* *Cyclist* *Pedestrian* *Fall* *Other*	25 (10) 45 (18) 50 (20) 9 (4) 83 (34) 34 (14)	22 (11) 35 (18) 38 (19) 4 (2) 73 (36) 29 (14)	2 (7) 5 (13) 8 (27) 4 (13) 8 (27) 3 (10)	1 (7) 5 (33) 4 (27) 1 (7) 2 (13) 2 (13)
High-energy trauma, N (%)	97 (39)	78 (39)	12 (40)	7 (47)
Crush injury, N (%)	17 (7)	12 (6)	2 (7)	3 (20)
ISS, median (IQR)	9 (4–9)	6 (4–9)	9 (4–10)	9 (4–11)
*Fracture characteristics*				
AO/OTA location, N (%) *Humerus* *Forearm* *Femur* *Tibia/fibula*	20 (8) 64 (26) 47 (19) 115 (47)	19 (10) 56 (28) 43 (21) 83 (41)	1 (3) 6 (20) 3 (10) 20 (67)	0 2 (13) 1 (7) 12 (80)
AO/OTA fracture type, N (%) *A 1 / 2 / 3* *B 1 / 2 / 3* *C 1 / 2 / 3*	8 (3) / 25 (10) / 22 (9) 19 (8) / 18 (7) / 40 (16) 10 (4) / 26 (11) / 78 (32)	7 (4) / 21 (10) / 19 (10) 14 (7) / 13 (7) / 37 (18) 5 (3) / 18 (9) / 67 (33)	0 / 3 (10) / 2 (7) 4 (13) / 3 (10) / 2 (7) 4 (13) / 5 (17) / 7 (23)	1 (7) / 1 (7) / 1 (7) 1 (7) / 2 (13) / 1 (7) 1 (7) / 3 (20) / 4 (27)
Soft tissue status (open), N (%)	60 (24)	35 (17)	15 (50)	10 (67)
Gustilo grade open fractures, N (%) *1* *2* *3 A / B / C*	18 (7) 20 (8) 9 (4) / 10 (4) / 3 (1)	12 (6) 13 (7) 6 (3) / 3 (2) / 1 (1)	10 (33) 5 (17) 3 (10) / 2 (7) / 1 (3)	2 (13) 2 (13) 0 / 5 (33) / 1 (7)

SD: standard deviation, IQR: interquartile range, BMI: Body Mass Index, ASA: American Society of Anesthesiologists physical status score, CCI: Charlson Comorbidity Index, ISS: Injury Severity Score, AO/OTA: Arbeitsgemeinschaft für Osteosynthesefragen/Orthopaedic Trauma Association, LOS: length-of-stay

### Primary outcome: direct healthcare costs

In patients with a single FRI occurrence after a long bone fracture, the baseline total direct healthcare costs were three (3.1) times higher compared to patients without FRI. The highest costs were seen in the FRI subgroup with recurrent infection, which is more than seven (7.6) times higher compared to non-FRI patients. Ratios per costs category are presented in Table 2.

**Table 2:** Outcomes per group: non-FRI, single FRI and FRI recurrence

Outcome	Non-FRI (*N* = 201)	Single FRI (*N* = 30)	FRI recurrence (*N* = 15)
Direct healthcare costs in ratios			
*Total costs, ratio*	***1.0***	***3.1***	***7.6***
Hospitalisation costs, ratio	1.0	5.0	12.6
Surgery costs, ratio	1.0	2.3	4.2
Consult costs, ratio	1.0	1.8	5.1
Imaging costs, ratio	1.0	2.1	3.1
Other costs, ratio	1.0	4.8	12.5
Healthcare utilisation			
Direct closure possible, N (%)	197 (98)	27 (90)	8 (53)
Additional surgeries (N), median (IQR)	0 (0–1)	2 (1–4)	7 (4–11)
Implant removal, N (%)	55 (27)	14 (47)	10 (67)
Total LOS (days), median (IQR)	5 (3–10)	25 (16–33)	50 (36–95)
Additional admissions (N), median (IQR)	0 (0–0)	1 (1–2)	3 (2–6)
Antibiotics costs (euros), median (IQR)	NA	765 (470 − 1.150)	1.350 (170 − 3.130)
Antibiotic duration (days), median (IQR) *Total (IV + oral)* *IV only*	NA NA	35 (23–43) 21 (10–41)	77 (22–114) 55 (35–126)
Follow-up period (years), median (IQR)	1.0 (0.5–1.6)	1.3 (0.8–2.2)	3.0 (1.8-4.0)

### Secondary outcome: healthcare utilisation

Table 2 presents outcomes regarding healthcare utilisation per group. Non-FRI patients in this study cohort underwent almost no additional surgeries (IQR 0–1), implant removal in 27.4% (*N* = 55) of cases and the total median LOS was five days (IQR 3–10). FRI patients without recurrence of their disease underwent on average two extra fracture related surgical procedures (IQR 1–4) and in 46.7% (*N* = 14) of cases implants were removed. The median total LOS was 25 days (IQR 16–33). Antibiotic treatment had a median duration of 35 days (IQR 23–43). In the subgroup with FRI recurrence the number of all outcomes increased dramatically, with a median of seven additional surgeries (IQR 4–11), total LOS of 50 days (IQR 36–95) and median duration of antibiotic treatment of 77 days (IQR 22–114).

## Discussion

This study showed that in case of an FRI, direct hospital healthcare costs increase up to three times and even more than seven times in case of recurrent FRI. To put these ratios in perspective, it should be noted that the health care and productivity costs of severely injured Dutch trauma patients was recently established at approximately 25.000 euros [15]. Our findings are in accordance with previous studies, confirming that FRI is associated with a high burden on healthcare expenditure [1, 5–8, 16, 17]. The previously reported increased costs varied from two up to eight times in infected patients. The lower limit of this increase is probably an underestimation, since these studies were considering unclassified infections [1] or infections classified based on the Centers for Disease Control and Prevention (CDC) criteria [16]. A recent study showed that the CDC definition had less diagnostic value and identified only half of all patients with an FRI. This is due to the short cut-off point (30 days) in which these complications are registered leading to an unrepresentative comparison [18]. On the other hand, Iliaens et al. (2021) reported, with eight times higher costs, the highest cost increase. Since their study excluded patients with other complications from analysis, their control group included a highly selected, relatively healthy group of patients with assumedly low overall costs. Therefore, this could have led to an overestimation of the difference in costs between patients with and without an FRI. Our study adds to previous reports because patients were selected according to the FRI consensus criteria and inclusion was regardless of other complications. Also, our study is the first to consider a subgroup of patients with infection recurrence.

Regarding healthcare utilisation, in our study single FRI patients had a fivefold prolonged total LOS compared to the non-FRI patients, mainly caused by a median of two extra surgical procedures and a median of 21 intravenous (i.v.) antibiotic treatment days. Patients receiving intravenous antibiotic treatment were discharged if there was no persistent wound leakage. For the subgroup of 15 patients with FRI recurrence the total LOS was prolonged tenfold, with a median of seven additional surgical procedures and a median of 55 days of i.v. antibiotic treatment. It is understandable that FRI patients require additional surgeries since current FRI treatment recommendations advise adequate debridement followed by adequate antibiotic therapy [19, 20]. Initial antibiotic therapy consists of i.v. administration for 7–10 days of a broad-spectrum antibiotic until pathogens and their antibiotic sensitivity profile are determined and the wound is sufficient to switch to oral antibiotics, for a total duration of 6–12 weeks depending on the presence of implants [21]. Both treatment pillars, additional surgeries and i.v. antibiotic therapy are probably the main cause of prolonged hospitalization, the known major cost driver of direct hospital expenses. In previous studies, a 1.2 to 10-fold longer LOS is described [1, 4, 5, 7, 17, 22]. Our findings add to previous literature because it reports on LOS in patients with and without FRI and demonstrated that there is a fivefold increase of LOS in FRI patients. Our findings showed that this is mainly due to FRI related extra surgical procedures and a median of 21 i.v. antibiotic treatment days. Also, this is to our knowledge the first study reporting on costs in case of FRI recurrence. Our study showed a rise of seven additional surgeries and a tenfold longer LOS in this subgroup compared to the non-FRI group. In current times, healthcare systems worldwide face significant challenges, marked by shortage of admission and treatment capacity and necessitating extensive cost-reducing measures. Therefore, the outcome of our study contributes to knowledge about the financial burden of FRI.

### Limitations

This study has several limitations. First, the FRI recurrence group was small and therefore all assumptions about patients with FRI recurrence should be interpreted with caution. On the other hand, it is one of the largest studies on costs associated with FRI treatment and fortunately FRI recurrence is less common in clinical practice. Secondly, since this is a retrospective study, no statement could be made regarding costs generated outside the hospital and indirect costs, such as inability to work. Also, our study included a relatively healthy population due to exclusion of patients with inseparable hospital expenses related to comorbidities. However, this should not affect the ratio between FRI and non-FRI. On the other hand, excluding hospital expenses related to comorbidities provided insight in the costs that should actually be attributed to FRI. Lastly, this study is conducted in a single-centre cohort. Since treatment might differ between different sites, it would be interesting to expand this study to multiple centers. 

## Conclusion

Direct healthcare costs of patients with single occurrence of FRI after long bone fracture treatment are three times higher compared to non-FRI patients. In case of FRI-recurrence, the differences in costs might even increase to sevenfold. To put this in perspective, costs of severely injured trauma patients were recently established at approximately 25.000 euros. Compared to non-FRI patients, increased costs in patients with FRI or recurrent FRI are due to respectively a fivefold or even tenfold prolonged length-of-stay, two or seven additional infection-related surgeries and 21 or 55 days of intravenous antibiotic treatment. Not only from patient perspective but also from a financial aspect, it is important to focus on prevention of (recurrent) FRI.

## Data Availability

No datasets were generated or analysed during the current study.
